# Editorial: The crosstalk of different mechanisms in cognitive impairment associated with aging, Alzheimer's disease, and related dementias

**DOI:** 10.3389/fnagi.2023.1258893

**Published:** 2023-08-18

**Authors:** Lydia Giménez-Llort

**Affiliations:** ^1^Institut de Neurociències, Universitat Autònoma de Barcelona, Barcelona, Spain; ^2^Department of Psychiatry and Forensic Medicine, School of Medicine, Universitat Autònoma de Barcelona, Barcelona, Spain

**Keywords:** Alzheimer's disease, cognitive impairment, crosstalk, peripheral system, clinical trials, translational research

## Introduction

In recent years, significant advances have been made in understanding the pathogenesis and treatment of Alzheimer's disease (AD). Growing evidence indicates that AD is a multifactorial and complex disorder, and its occurrence and development are influenced by multiple pathogenic processes rather than a single factor (Turrini et al., 2023). Although several mechanisms of AD pathogenesis, such as cholinergic neuron damage, amyloid β (Aβ), tau, oxidative stress, mitochondrial dysfunction, inflammation, and glucose hypometabolism, have been revealed, the effective treatment of AD remains elusive (Reiss et al., 2023). Under an integrative systems research approach, the brain is investigated in interactions with peripheral organs. Yet, the role of their crosstalk in the cognitive impairments associated with aging, Alzheimer's disease, and related dementias remains unclear (Giménez-Llort et al., 2012, 2014). Therefore, this Research Topic encouraged pre-clinical (basic science) and clinical research to explore the crosstalk of different pathogenic mechanisms and/or different tissues in aging and AD-related cognitive impairments.

## On the role of type 2 diabetes mellitus

The mechanisms and implications underlying cognitive decline, mild cognitive impairment (MCI), and dementia in Type 2 diabetes mellitus (T2DM) have attracted significant attention in recent years since a large number of studies have found that T2DM may increase their incidence rate (Biessels and Despa, 2018; Husain et al., 2023). In this Research Topic, two works addressed interesting aspects of this issue. First, a systematic review by Long et al. on the potential efficacy of intranasal (IN) insulin in improving cognitive function in people with MCI/dementia, without exerting undesirable peripheral hypoglycemia (Craft et al., 1996, 2020), critically assessed 16 randomized controlled clinical trials gathering a total of 899 patients. The analysis depicted the number and patients' characteristics and diagnosis, acute/chronic treatment regimens and duration, therapeutic device, type and dose of insulin, cognitive test used, potential mechanisms of intranasal insulin in MCI or dementia treatment, and adverse effects. The combined cognitive performance score change did not differ between IN insulin and placebo groups. However, an improvement in cognitive outcomes in the Alzheimer's Disease Cooperative Study-activities of daily living (ADCS-ADL) was found when results were specifically examined per cognitive test. Interestingly, IN insulin improved verbal memory performance but no other cognitive functions in APOe4 (-) people. Despite limitations, the systematic review indicated the promising potential of intranasal insulin and advised on the need for study designs using larger doses and considering the insulin type. The effect of the APOE genotype points to the relevance of the group chosen to carry out the study on, while the study was also in line with recent data on a subgroup of dementia related to specific DM-associated metabolic abnormalities (Hanyu, 2019).

In the second work, the original clinical research by Cao et al., a cross-sectional study, explored the role of inflammation in type 2 diabetes mellitus comorbid with major depressive disorder (MDD) and the cognitive impairments and clinical symptoms associated with these disorders. In particular, the authors assessed monocyte chemoattractant protein-1 (MCP-1), a small, signaling protein secreted by cells of the immune system that triggers chemotaxis and the transendothelial migration of monocyte to the inflammatory areas (Singh et al., 2021). Serum MCP-1 was able to diagnose T2DM with high sensitivity and specificity, appearing as a promising biomarker of T2DM. It also exhibited high discriminatory capacity in the group with comorbidities, with foreseeable potential value for early clinical prediction or diagnosis of cognitive impairment in people with T2DM and MDD comorbidity. Moreover, this work also provided further evidence that comorbidity aggravated cognitive dysfunction. In addition, the cognitive impairment correlated with the worsening of T2DM and DMM hallmark symptoms, suggesting that MCP-1 may be involved in the complex mechanisms underlying comorbid scenarios; this deserves further clinical and pre-clinical studies.

## On the role of peripheral immune function

Two translational research approaches, using well-known double- and triple-transgenic murine models for Alzheimer's disease, addressed the peripheral immune function in the disease at early and very advanced stages, respectively. In the first translational approach, gut microbiomics (16S rRNA gene sequencing of fecal samples) and fecal metabolomics (widely targeted UPLC-MS/MS metabolomics) analyses were used by Feng et al. to characterize gut bacterial communities and fecal metabolic profiles in 7-month-old (early stages) male APP/PS1 mice showing learning deficits as compared to age-matched wild-type mice when assessed in the Morris water maze. On the one hand, ß-diversity was reduced due to a higher ratio of *Firmicutes/Bacteroidota* and multiple differential bacteria. Afterward, pathway analysis of differential metabolites identified a high proportion of metabolites classified into metabolism, and a significant perturbation of pyrimidine metabolism, with depletion of deoxycytidine, 2′-deoxyuridine, and thymidine in APP/PS1 mice. On the other hand, *B. firmus, Rikenella, Clostridium* sp. Culture-27, and deoxyuridine were identified as biological markers, playing an important role in early AD. Although biological validation of these findings needs to be done, the involvement of gut dysbiosis in the disruption of pyrimidine metabolism in this animal model encourages the investigation of this pathological pathway in clinical trials. In summary, the work contributes to unveiling the interplay of the host-gut microbiota crosstalk and microbiome-related metabolites in the pathogenesis of the disease, useful as diagnostic biomarkers and therapeutic targets.

The second translational neuroscience scenario investigated innate immune system derangements in Alzheimer's disease, resulting in crosstalk with neuronal and endocrine functions. To investigate the crosstalk between cognitive and neuropsychiatric-like symptoms, HPA axis, splenic oxidative stress, and frailty, 18-month-old female 3xTg-AD mice surviving beyond advanced stages of Alzheimer's disease (AD) and age-matched non-transgenic counterparts with normal aging were used (Muntsant and Giménez-Llort). AD neuropsychiatric-like profiles and cognitive dysfunction persisted despite losing other behavioral and hypothalamic-pituitary-adrenal (HPA) physiological differences. Social isolation, naturally occurring due to the death of cage mates, disrupted the obsessive-compulsive disorder-like ethogram, and modified hyperactivity and neophobia patterns. In all groups, spleen organometrics correlated with the frailty index; however, AD-specific salient functional correlations (corticosterone levels with worse memory performance and lower splenic GPx antioxidant enzymatic activity, a potent risk of morbidity and mortality in AD) were identified.

The present works contribute to identifying the crosstalk between different mechanisms, better understanding the brain, and providing insights into novel preventive/therapeutical strategy targeting.

## Author contributions

LG-L: original idea and writing—original draft preparation, editorial work and conceptualization. The author confirms being the sole contributor of this work and has approved it for publication.
